# Encéphalite limbique paranéoplasique compliquant un adénocarcinome pulmonaire: à propos d’un cas

**DOI:** 10.11604/pamj.2021.39.95.26568

**Published:** 2021-06-02

**Authors:** Houda Snene, Khalil Zayen, Nozha Ben Salah, Hana Blibech, Leila Ben Farhat, Aïda Ayadi, Saoussen Hantous, Nadia Mehiri, Béchir Louzir

**Affiliations:** 1 Université de Tunis El Manar, Faculté de Médecine de Tunis, Centre Hospitalier Universitaire Mongi Slim, La Marsa, Service de Pneumologie Allergologie, Tunis, Tunisie,; 2 Université de Tunis El Manar, Faculté de Médecine de Tunis, Centre Hospitalier Universitaire Mongi Slim, La Marsa, Service de Radiologie, Tunis, Tunisie,; 3 Université de Tunis El Manar, Faculté de Médecine de Tunis, Centre Hospitalier Universitaire Abderrahmen Mami, Service d´Anatomie Pathologie, Ariana, Tunisie,; 4 Université de Tunis El Manar, Faculté de Médecine de Tunis, Centre Hospitalier Universitaire Abderrahmen Mami, Service de Radiologie, Ariana, Tunisie

**Keywords:** Adénocarcinome pulmonaire, encéphalite limbique, syndrome neurologique paranéoplasique, à propos d’un cas, Pulmonary adenocarcinoma, limbic encephalitis, paraneoplastic neurological syndrome, case report

## Abstract

L´encéphalite limbique (EL) est une pathologie rare souvent d´origine paranéoplasique avec une association fréquente au cancer broncho-pulmonaire. Son diagnostic est radiologique, reposant sur l´imagerie par résonnance magnétique (IRM) cérébrale. Nous rapportons le cas d´une femme âgée de 54 ans, tabagique, qui consulte pour toux sèche trainante. La radiographie du thorax avait montré une opacité pulmonaire droite suspecte. La fibroscopie bronchique et les biopsies bronchiques n´étaient pas contributives. Au scanner thoraco-abdomino-pelvien et cérébral, il existait une masse du lobe supérieur droit classée T4N2M1a. Une biopsie pulmonaire scanno-guidée a confirmé le diagnostic d´un adénocarcinome broncho-pulmonaire. Au cours de son exploration, la patiente avait rapporté la notion de troubles récents de la mémoire avec une humeur dépressive, une anxiété et une confusion paroxystique. Les bilans métaboliques et infectieux étaient normaux et l´IRM cérébrale était en faveur d´une EL. L´évolution a été rapidement progressive et la patiente est décédée en une dizaine de jours.

## Abstract (en)

Limbic encephalitis (LE) is a rare disease often of paraneoplastic origin. It is frequently associated with bronchopulmonary cancer. Diagnosis is based on brain magnetic resonance imaging (MRI). We here report the case of a 54-year-old female patient with a history of active smoking, presenting with chronic dry cough. Chest X-ray showed suspicious right lung opacity. Bronchial fibroscopy and bronchial biopsies were not contributory. Thoraco-abdomino-pelvic computed tomography (CT)-scan and brain CT-scan showed a mass in the right upper lobe classified as T4N2M1a. CT-guided lung biopsy confirmed the diagnosis of bronchopulmonary adenocarcinoma. The patient had reported a recent history of memory disturbances associated with depressed mood, anxiety and paroxysmal confusion. Metabolic screening and tests for detecting infection were normal and brain MRI suggested limbic encephalitis. The evolution was characterized by rapid disease progression; the patient died in about ten days.

## Introduction

L´encéphalite limbique est une pathologie rare correspondant à des atteintes inflammatoires et immunologiques de l´encéphale portant ou prédominant sur le grand lobe limbique comme décrit par Paul Broca en 1878 [1]. L´origine paranéoplasique en serait l´une des étiologies. L´association avec un cancer pulmonaire est la plus fréquente particulièrement le carcinome neuroendocrine à petites cellules [2]. Son diagnostic clinique repose sur la survenue aiguë ou subaiguë d´un tableau associant épilepsie temporale, troubles de la mémoire et troubles psychiatriques dans des proportions variables (syndrome dépressif, irritabilité, troubles du comportement ou délire avec hallucinations) [3]. Le diagnostic repose sur l´imagerie par résonnance magnétique qui montre des anomalies de signal temporo-limbique [4]. Les avancés en immunologie ont permis l´identification de plusieurs anticorps appelés « anticorps anti-onconeuronaux » qui restent absents dans 40% des cas [5]. Le pronostic est sombre et le seul traitement curatif est la résection précoce de la tumeur primitive [6]. Nous rapportons le cas de notre patiente vue l´association de son EL à un carcinome pulmonaire non à petites cellules et surtout vue la symptomatologie clinique déroutante sans épilepsie associée et l´évolution rapidement progressive vers le décès.

## Patient et observation

**Information de la patiente:** une femme âgée de 54 ans, infirmière, tabagique à 60 paquet-année (PA) non sevrée, consulte pour une toux sèche évoluant depuis deux mois, non-améliorée par les traitements symptomatiques.

**Résultats cliniques:** à l´examen physique, on trouvait un Performance Status à 0 et un hippocratisme digital.

**Démarche diagnostique:** la radiographie du thorax avait montré une opacité pulmonaire, hilo-axillaire droite, grossièrement arrondie, à contours spiculés, associée à une opacité para-cardiaque gauche de contours flous. A la fibroscopie bronchique, il existait un élargissement des éperons des sous-segmentaires de la bronche dorsale de la lobaire supérieure droite, qui ont été biopsiés. L´examen anatomopathologique ne retrouvait pas de signes de malignité, cependant, l´examen cytologique du liquide bronchique était suspect d´un adénocarcinome (il y a été observé des amas tridimensionnels de grandes cellules atypiques avec de gros noyaux à limites irrégulières, qui sont par places vacuolisées). A la tomodensitométrie thoraco-abdomino-pelvienne et cérébrale, il existait une masse du segment dorsal du lobe supérieur droit de 60mm de grand axe, au contact des deux scissures qu´elle semblait franchir, associée à un nodule de 13mm adjacent à son pôle supérieur et une lymphangite carcinomateuse touchant les trois lobes. Il existait aussi des adénomégalies nécrosées hilaires homolatérales (10R) et médiastinales (2R, 4R et 7) et une lésion secondaire du segment latéro-basal du lobe inférieur gauche de 30mm de grand axe sans autres localisations métastatiques particulièrement cérébrales, classant la tumeur en T4N2M1a (stade IV). Une biopsie pulmonaire transpariétale scanno-guidée a été indiquée et a confirmé le diagnostic d´un adénocarcinome dont le profil immune-histochimique était en faveur de son origine broncho-pulmonaire. Au cours de son hospitalisation pour la biopsie pulmonaire, le fils de la patiente avait rapporté la notion de troubles récents de la mémoire chez sa mère avec des propos incohérents par moment. A l´examen, elle avait une humeur dépressive, était anxieuse et paraissait confuse dans certaines situations.

**Figure 1:**
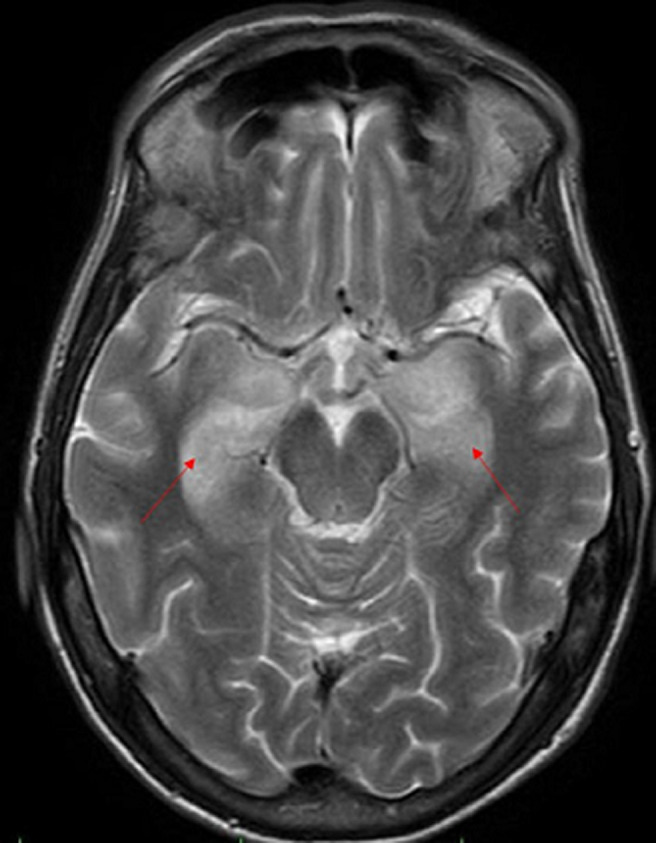
coupe axiale d’une IRM cérébrale montrant un hypersignal T2, en cortico-sous cortical temporal interne bilatéral

**Figure 2:**
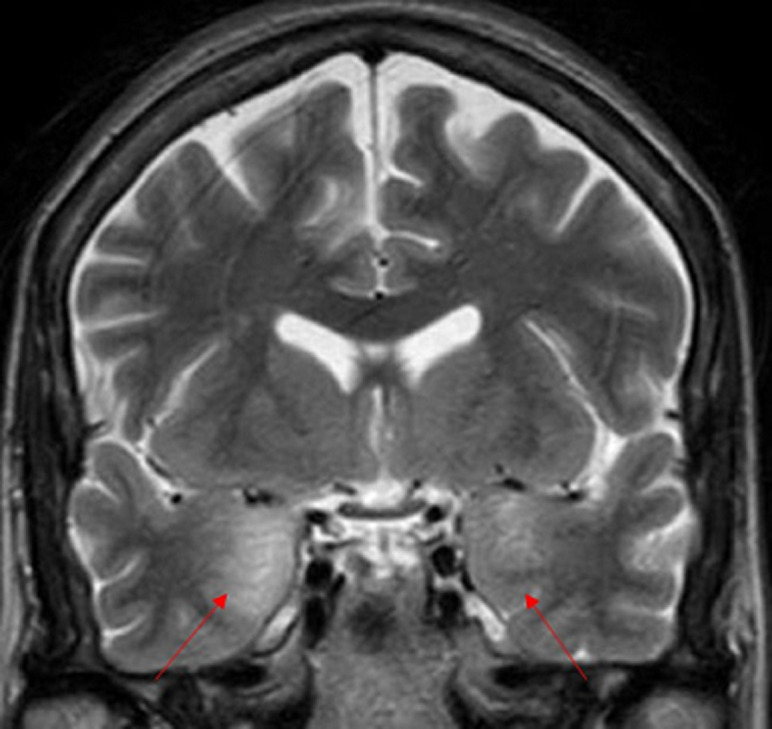
coupe coronale d’une IRM cérébrale montrant un hypersignal T2, en cortico-sous cortical temporal interne bilatéral

**Figure 3:**
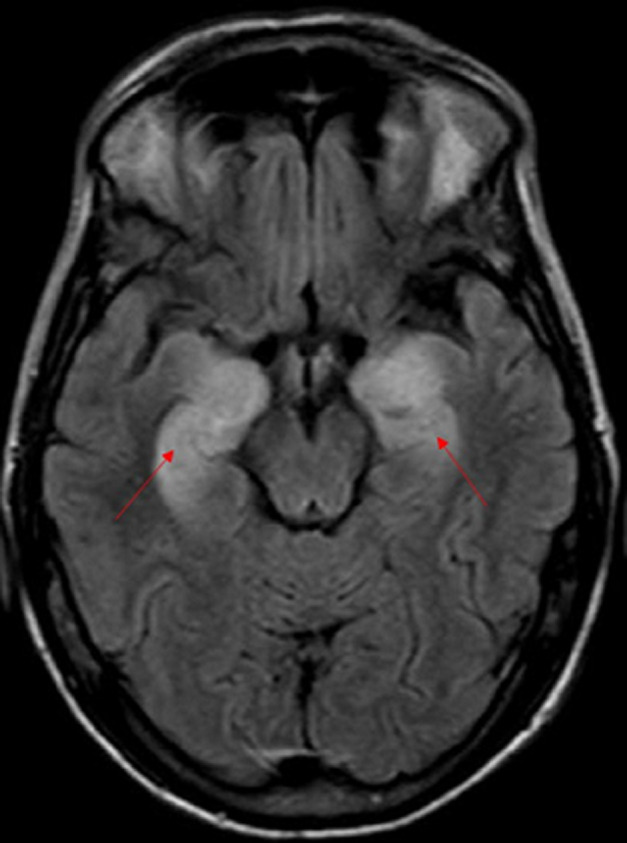
coupe axiale d’une IRM cérébrale montrant un hypersignal T2-FLAIR, en cortico-sous cortical temporal interne bilatéral

Les bilans métaboliques et infectieux étaient normaux en particulier pas de troubles ioniques. L´analyse du liquide céphalorachidien a montré une légère élévation des protides (0,51 g/l), sa culture en milieux bactériologiques usuels n´a pas mis en évidence de germes (la recherche du virus herpès simplex n´a pas été réalisée) et il n´y avait pas été observé de cellules tumorales. L´imagerie par résonnance magnétique (IRM) cérébrale était en faveur d´une EL (Figure 1, Figure 2 et Figure 3). Un dosage des anticorps anti-onconeuronaux a été indiqué mais non fait (non disponible à notre hôpital). L´électro-encéphalogramme a été programmé (dans un autre centre hospitalier car non disponible à notre hôpital).

**Intervention thérapeutique et suivi:** malheureusement l´évolution a été rapidement progressive et la patiente est décédée au bout d´une dizaine de jours alors qu´elle était en attente de l´accord des caisses nationales d´assurance maladie pour sa chimiothérapie. Les différentes investigations réalisées pour exclure les diagnostics différentiels sont résumées dans le Tableau 1.

**Tableau 1:** diagnostics différentiels de l´encéphalite limbique paranéoplasique

Diagnostic différentiel	Elément recherché pour l´exclure
**Traumatisme crânien**	Pas de traumatisme rapporté à l´interrogatoire
**Maladies dégénératives (Alzheimer, démence fronto-temporale)**	Les troubles mnésiques sont récents. IRM: pas d´atrophie des lobes temporaux ni de l´hippocampe.
**Causes infectieuses:**	
Méningo-encéphalite herpétique	Pas de fièvre ni céphalées. A l´IRM: pas de perte de la différentiation entre le cortex et la substance blanche et les anomalies de signal cortico-sous corticales sont bilatérales.
Neurosyphilis	Pas d´antécédent de syphilis primaire. Pas d´hyperexcitabilité ou de délire mégalomaniaque. A l´IRM: les anomalies de signal sont symétriques. Pas de dilatation quadri ventriculaire. Pas d´atrophie corticale, sous corticale ou des lobes frontaux, temporaux ou pariétaux.
**Causes métaboliques:**	
Troubles endocriniens (Cushing)	Pas d´hypokaliémie ni hyperglycémie ni alcalose.
Encéphalite de Gayet Wernicke	IRM: pas d´hypersignal FLAIR des tubercules mamillaires ni en péri aquaducal.
**Maladies systémiques:**	
Lupus érythémateux systémique	Pas de lésions cutanées ni anémie ni lymphopénie. Pas d´atteinte articulaire. IRM: les anomalies à l´IRM ne sont pas en faveur.
Syndrome de Sjögren	Pas de syndrome sec ni atteinte articulaire. IRM: les anomalies à l´IRM ne sont pas en faveur.
Encéphalite de Hashimoto et SREAT	La thyroïde est de taille normale à la palpation. IRM: les anomalies à l´IRM ne sont pas en faveur.
**Causes vasculaires**	IRM: Pas de lésions d´infarcissement.
**Epilepsie temporale**	Pas de crise d´épilepsie rapportée à l´interrogatoire. IRM: pas de malformation hippocampique. EEG: programmé mais non fait.

**IRM:** imagerie par résonnance magnétique; EEG: électro-encéphalogramme

## Discussion

L´EL paranéoplasique a été décrite pour la première fois en 1968 [7]. Elle peut précéder le diagnostic de cancer dans 60% des cas avec un délai moyen de 3,5 mois. Dans le cas de notre patiente, le diagnostic de l´EL a été fait au moment de l´exploration de la néoplasie. Les néoplasies auxquelles elle s´associe sont le cancer broncho-pulmonaire dans 50% des cas, les tumeurs testiculaires dans 20% des cas et le cancer du sein dans 8% des cas [2]. Les tumeurs broncho-pulmonaires sont dans la majorité des cas des carcinomes neuroendocrines à petites cellules (40% à 54% des cas) tandis que les cancers non à petites cellules (CNPC) ne sont retrouvés que dans 4 à 10% des cas [2]. Dans le cas que nous rapportons, il s´agit d´un adénocarcinome broncho-pulmonaire. Il s´agit d´une association rare tel que rapporté par Shahani [8] et Morelli-Zaher [9] dans leurs cas cliniques respectifs, où l´EL a révélé un adénocarcinome pulmonaire. Les mécanismes physiopathologiques de cette atteinte ne sont que partiellement élucidés. L´augmentation d´auto-anticorps dans le sang et dans le liquide céphalo-rachidien dirigé contre le tissu nerveux suggère un phénomène d´auto-immunité et la baisse du taux de ces anticorps après traitement de la tumeur ne fait que réconforter cette hypothèse. Les principaux anticorps anti-onconeuronaux recherchés sont: anti-Hu, anti-CV2/CRMP5, anti-amphiphysine, anti-Ma1 et anti-Ma2, anti-Tr et anti-Yo. Les anticorps les plus fréquemment retrouvés sont les anti-Hu dans 50% des cas [3]. Ils sont retrouvés en cas de tumeurs broncho-pulmonaires type carcinome neuroendocrinien à petites cellules, d´adénocarcinome du colon, de thymomes et de tumeurs broncho-pulmonaires type carcinome pulmonaire non à petites cellules [10-13]. Les autres anticorps détectés en cas de tumeurs broncho-pulmonaires type carcinome pulmonaire non à petites cellules sont les anti-Ma2 [14].

Sur le plan macroscopique, l´encéphale est normal. Histologiquement, on retrouve une perte neuronale, une infiltration lymphocytaire péri-vasculaire et une gliose réactionnelle. Après la phase inflammatoire, apparait la phase cicatricielle irréversible et atrophique. Ces lésions prédominent sur la substance grise du cortex, la partie interne des lobes temporaux, la région de l'hippocampe et les noyaux amygdaliens [4]. La symptomatologie est faite de crises d´épilepsie temporale, de troubles de la mémoire antérograde et/ou de troubles psychiatriques à type de syndrome dépressif, d´irritabilité, de troubles du comportement ou de délire avec hallucinations [3]. Ces symptômes peuvent mimer les manifestations cliniques des métastases cérébrales, ceux des effets secondaires de la chimiothérapie ou ceux des troubles métaboliques. Toutefois, leur caractère aigu ou subaigu et l´absence de signes d´hypertension intracrânienne et de troubles métaboliques doivent faire évoquer ce diagnostic. Chez notre patiente, la symptomatologie était dominée par des troubles amnésiques, une humeur dépressive, une anxiété et une confusion paroxystique. Par ailleurs, il existe une classification en fonction du type d´anticorps qui permet l´identification de syndromes cliniques, d´une histoire naturelle et d´une réponse au traitement spécifique à chaque anticorps [3]. Enfin, 20% des EL séronégatives ne peuvent encore être classées [2].

Le scanner cérébral est souvent normal. L´IRM montre un hypersignal T2 et T2-FLAIR (récupération d’inversion atténuée par le liquide) impliquant les structures temporo-mésiales de manière uni ou bilatérale sans effet de masse ni prise de contraste après injection de Gadolinium [3]. Elle doit être réalisée dans le plan bihippocampique afin de mettre en évidence des lésions temporo-mésiales parfois discrètes [3]. Ces anomalies apparaissent après un certain délai et l´IRM peut être normal au début [3, 4]. Le caractère paranéoplasique ne peut être retenu qu´après élimination des causes infectieuses, métaboliques et toxiques. Le dosage des anticorps anti-onconeuronaux peut contribuer au diagnostic. Dans le cas que nous rapportons, il existait un hypersignal T2-Flair cortico-sous corticale temporal interne bilatéral intéressant l´hippocampe, le gyrus para-hippocampique et les amygdales.

Le traitement de ces EL d´origine paranéoplasique repose avant tout sur le traitement du cancer associé. Il existe dans la majorité de ces cas des anticorps à cible intracellulaire et le traitement immunomodulateur (immunoglobulines intraveineuses, échanges plasmatiques et corticoïdes) sera peu efficace et les séquelles neurologiques seront souvent définitives. Seul le cyclophosphamide est proposé en cas de signe d´évolutivité neurologique en dépit d´un traitement oncologique optimal [3]. Le pronostic immédiat est lié à la localisation et à l´étendu de l´inflammation pouvant occasionner un coma, des troubles neurovégétatifs ou même le décès. A plus long terme, le pronostic est lié au délai de la prise en charge de la tumeur. Dans notre cas, l´évolution a été rapidement progressive et la patiente est décédée avant même d´avoir bénéficiée d´une chimiothérapie.

## Conclusion

L´EL est rare et l´origine paranéoplasique en est la cause la plus fréquente. Elle est souvent liée à un carcinome neuroendocrinien à petites cellules pulmonaire mais les carcinomes pulmonaires non à petites cellules ont été rapportés tel notre cas. Son approche diagnostique a été bouleversée par les découvertes dans le domaine de l´immunologie mais son diagnostic positif repose sur l´IRM cérébrale. Il faut savoir y penser devant toute symptomatologie psychiatrique associée à des troubles mnésiques apparus de façon aiguë ou subaiguë. Son traitement repose sur celui de la néoplasie associée mais son pronostic reste sombre.
